# Supplementary material for: Self-Powered Gradient Hydrogel Sensor with the Temperature-Triggered Reversible Adhension
Source: Polymers (Basel). 2022 Dec 5;14(23):5312. doi: 10.3390/polym14235312 (PMC9737204; doi:10.3390/polym14235312)
Supplement: Supplementary file 1 [file polymers-14-05312-s001.zip › polymers-2049936-supplementary.pdf]

Supporting Information

Self-powered gradient hydrogel sensor with the temperature-triggered reversible adhension

Dong Sun[1], Cun Peng[2], Yuan Tang[1], Pengfei Qi[1], Wenxin Fan[1], Qiang Xu[3,*] and Kunyan Sui[1*]

[1] State Key Laboratory of Bio-Fiber and Eco-Textiles, Collaborative Innovation Center for Marine Biobased Fibers and Ecological Textile Technology Institute of Marine Biobased Materials, College of Materials Science and Engineering, Qingdao University, Qingdao 266071, China

[2] College of Chemistry and Bioengineering, Hunan University of Science and Engineering, Yongzhou 425199, China

[3] Department of Ophthalmology, The Affiliated Hospital of Qingdao University, Qingdao 266071, China

* Correspondence: yankexuqiang@126.com (Q.X.); sky@qdu.edu.cn (K.S.)

Supplemental Figures

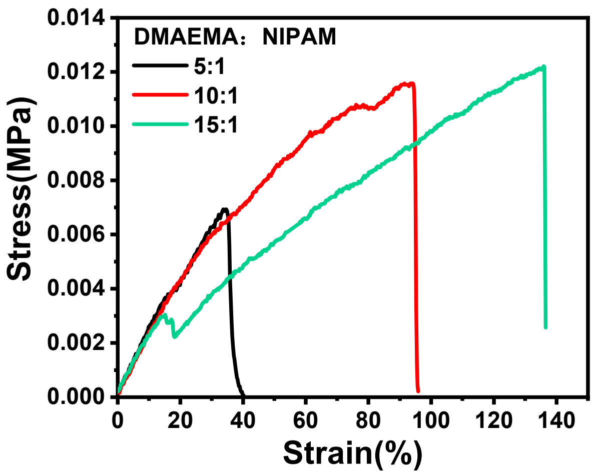

Figure S1. Stress-strain curves of the hydrogel with different DMAEMA/NIPAM mass ratios after 6 hours of UV irradiation.

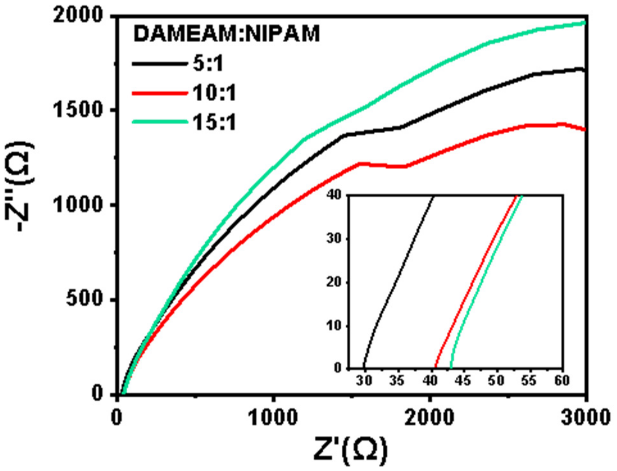

Figure S2. EIS Nyquist diagram of hydrogels membranes with different DMAEMA: NIPAM mass ratios.

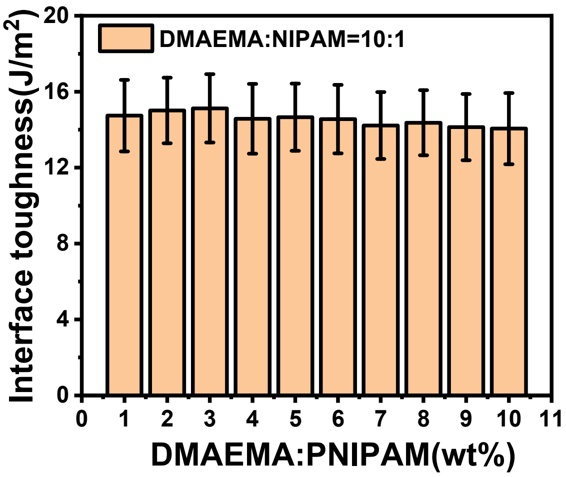

Figure S3. DMAEMA: NIPAM=10:1 interface toughness of the hydrogel after repeated adhesion of silicone rubber for 10 times.

Table S1. Summary of various self-powered sensors.

	Materials	Advantages	Disadvantages	Sensitivity
Piezoelectric sensors	PS/PDMS[1]	Suitable for micro-systems	Interface incompatibility	59.4mV/kPa
	PZT/PVDF[2]	High output voltage	Poor stability	6.38mV/N
	ZnO[3]	Self-power ability	Static sensing	0.62V/kPa
	P(VDF-TrFE)[4]	High power density	Electronic signal transmission	1.4V/kPa
TENG	AC/PU[5]	Miniaturization and lightness	Environment interferences	0.94V/kPa
	PTFE[6]	High conversion efficiency	Limited durability	——
Our work	PDMAEMA/ PNIPMA	Ion signal transmission<br>Accurately sense static pressure<br>Reversible adhesion		106.46mV/MPa

References

1. M. Mariello, L. Fachechi, F. Guido, M. D. Vittorio, Conformal, Ultra-thin Skin-Contact-Actuated Hybrid Piezo/Triboelectric Wearable Sensor Based on AlN and ParyleneEncapsulated Elastomeric Blend, Advanced Function Materials. 31 (2021) 2101047.
2. G. Tiana , W. Denga, Y. Gaoa, D. Xionga, C. Yana, X. Hea, T. Yanga, L. Jina, X. Chua, H. Zhanga, W. Yanb, W. Yang, Rich lamellar crystal baklava-structured PZT/PVDF piezoelectric sensor toward individual table tennis training, Nano Energy. 59 (2019) 574-581.
3. Y. Tan, K. Yang, B. Wang, H. Li, L. Wang, C. Wang, High-performance textile piezoelectric pressure sensor with novel structural hierarchy based on ZnO nanorods array for wearable application, Nano Research. 14 (2021) 3969-3976.
4. Y. Shin, S. Sohn, H. Han, Y. Park, H. Shin, H. Ko, Self-powered triboelectric/pyroelectric multimodal sensors with enhanced performances and decoupled multiple stimuli, Nano Energy. 72 (2020) 104671.
5. P. Yang, Y. Shi, S. Li, X. Tao, Z. Liu, X. Wang, Z. Wang, X. Chen, Monitoring the degree of comfort of shoes in-motion using triboelectric pressure sensors with an ultrawide detection range, ACS Nano. 16 (2022) 4654-4665.
6. D. Wang, D. Zhang, Y. Yang, Q. Mi, J. Zhang, L. Yu, Multifunctional latex/ polytetraflfluoroethylene-based triboelectric nanogenerator for self-powered organ-like MXene/metal-organic framework-derived CuO nanohybrid ammonia sensor, ACS Nano. 15 (2021) 2911-2919.
